# Supplementary material for: Test-retest reliability of task-based and resting-state blood oxygen level dependence and cerebral blood flow measures
Source: PLoS One. 2018 Nov 8;13(11):e0206583. doi: 10.1371/journal.pone.0206583 (PMC6224062; doi:10.1371/journal.pone.0206583)
Supplement: S1 File — (DOCX) [file pone.0206583.s001.docx]

**Supplementary Materials, Holiga et al.**

## Supplementary methods

**Table A** Study MRI assessments

|  |  | **ID** | **MRI measure** |  | **Domain** |  | **Duration** |  | **Screening** |  | **Visit 1** |  | **Visit 2** |
| --- | --- | --- | --- | --- | --- | --- | --- | --- | --- | --- | --- | --- | --- |
|  |  |  |  |  |  |  |  |  |  |  |  |  |  |
| **structure** |  | 1 | *T_1_*-w |  | - |  | 10m 14s |  | × |  |  |  |  |
|  |  | 2 | *T_2_*-w |  | - |  | 2m 52s |  | × |  |  |  |  |
|  |  | 3 | FLAIR |  | - |  | 3m 31s |  | × |  |  |  |  |
|  |  |  |  |  |  |  |  |  |  |  |  |  |  |
| **rest** |  | 4 | pCASL |  | - |  | 8m 39s |  | × |  | × |  | × |
|  |  | 5 | rs-fMRI BOLD |  | - |  | 8m 20s |  | × |  | × |  | × |
|  |  |  |  |  |  |  |  |  |  |  |  |  |  |
| **task-based** |  | 6 | Face Matching |  | Emotion processing |  | 5m 08s |  |  |  | × |  | × |
|  |  | 7 | Monetary Incentive Delay |  | Reward anticipation |  | 12m 18s |  |  |  | × |  | × |
|  |  | 8 | N-Back |  | Working memory |  | 4m 21s |  |  |  | × |  | × |
|  |  | 9 | Go/No-go |  | Response inhibition |  | 08m 20s |  |  |  | × |  | × |
|  |  | 10 | Episodic Memory |  | Encoding, recall and recognition |  | 16m 31s |  |  |  | × |  | × |
|  |  | 11 | Theory of Mind |  | Social cognition |  | 8m 6s |  |  |  | × |  | × |
|  |  |  |  |  |  |  |  |  |  |  |  |  |  |

Visit 1 and Visit 2 were fourteen days apart. The episodic memory task consisted of three parts (Encoding, Recall, Recognition). BOLD: blood oxygenation level-dependent, pCASL: pseudo-continuous arterial spin labeling, rs-fMRI: resting-state fMRI, *T_1_*-w: *T_1_*-weighted, *T_2_*-w: *T_2_*-weighted.

## Description of the fMRI tasks

The images were collected during rest or task execution, as described below. Before the onset of each new task, a pre-scan reminder with the illustration and a short description of the task was presented to the subject to ensure task understanding. Also, a predefined task explanation read word-by-word was presented to the subject by the operator via the intercom.

### pCASL and rs-fMRI

Subjects were instructed to “rest” in the scanner, not think about anything in particular, move as little as possible and not fall asleep while watching a fixation cross on the projection screen.

### Face Matching

The face matching task was adopted from Hariri et al. ([Hariri et al., 2002](#_ENREF_3)). The task was divided into nine blocks, four blocks of matching faces with fearful/angry facial expressions taken from the Ekman Pictures of Facial Affect stimulus set and five blocks of matching simple forms, i.e., circles and ellipses, as a sensorimotor control condition (Fig A). Each block contained six matching trials (5 s each), and for each trial, participants indicated which of the two bottom images, left or right, matched the center top image, by pressing the left or right button, respectively, on a button pad using the right thumb. Instructions were displayed for 2 s at the beginning of each block: “Match Faces” or “Match Forms.”


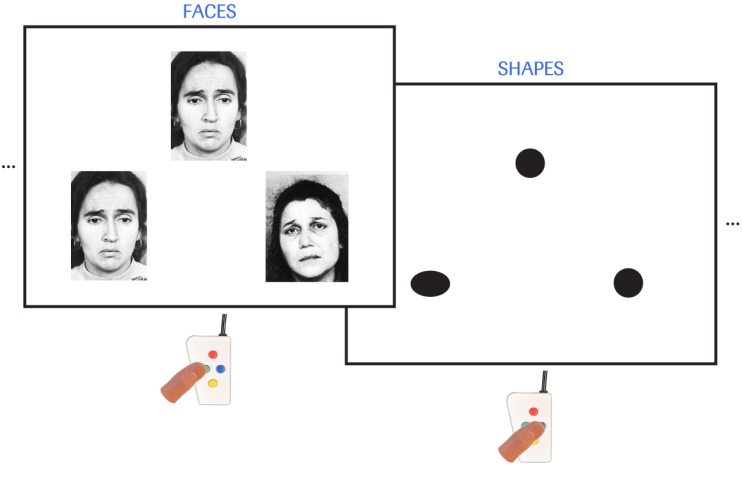


**Fig A.** **Face matching task**

### Monetary Incentive Delay

The task was adopted from Knutson et al. ([Knutson et al., 2008](#_ENREF_4)). The subjects were asked to respond as quickly as possible to a white box on the visual display screen. The box presentation was preceded by a stimulus that informed the subject about the consequences of their response to the stimulus itself. Three conditions were included in the paradigm: (1) Win-high condition: the subject ‘wins’ a higher amount of money if the response is sufficiently fast; (2) Win-low condition: the subject ‘wins’ a lower amount of money if the response is sufficiently fast; (3) Neutral control condition: the subject ‘wins’ or ‘loses’ no money but is still asked to respond as fast as possible (Fig B). At the end of each trial, a feedback on the total amount of money won as well as won in the last trial was presented. Accuracy, reaction time and amount of money gained were recorded. The reaction time window was adaptively tailored to the individual response times of the subject in order to have comparable winnings across subjects and sessions.


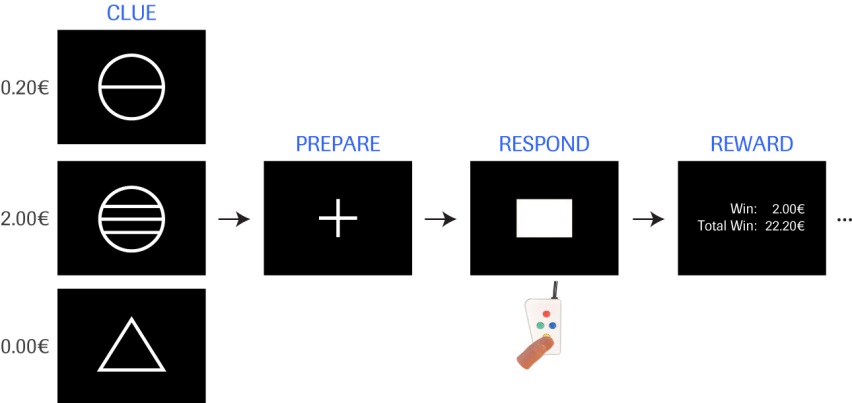


**Fig B.** **Monetary incentive delay task**.

### N-back

The N-back task of Callicott et al. was used ([Callicott et al., 1999](#_ENREF_1)). This task required subjects to constantly update their mental set while recalling previous stimuli. Numbers from 1-4 were continually presented at set points of a diamond, one at a time every 2 seconds for 500 ms. Subjects were instructed to press the button on a response box corresponding to the number seen in “current trial - n”, and were tested for 0- and 2-back memory loads (Fig C). Therefore, each number was a probe as well as a target. A block design was used, in which the 0-back (a sensory-motor control condition not involving working memory) alternated with the 2-back task for 30 seconds each, over 8 repetitions. Performance was measured as percent accuracy and reaction time.


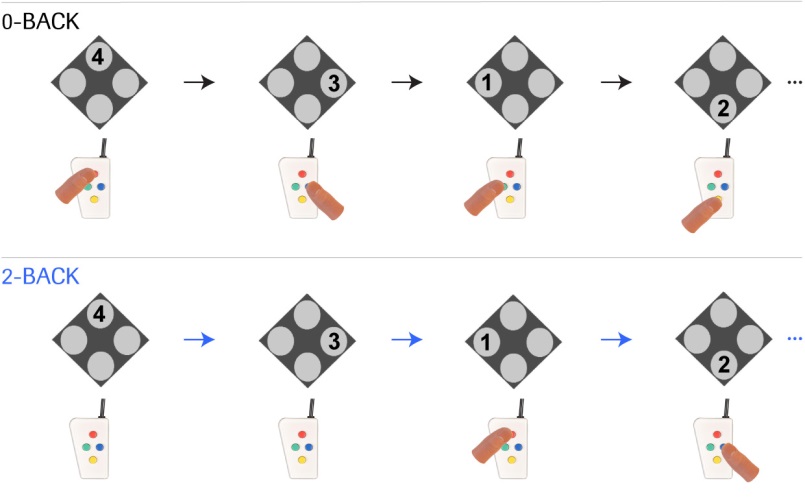


**Fig C.** **N-back task**.

### Episodic memory

The task was adopted from Erk et al. ([Erk et al., 2011](#_ENREF_2)). The subjects were asked to complete an episodic memory task with three components: encoding, recall, and recognition of face-profession pairs; Fig D. During recall, faces were presented together with the question whether the depicted person had completed an apprenticeship or academic studies to qualify for their respective profession. Subjects indicated the correct response by button press. In the control condition, subjects assessed whether the left or right ear of head contours was larger. The task consisted of four blocks of four faces (presented for 6 sec each) and four blocks of six head contours (presented for 4 sec each) respectively. Each block lasts 24 sec. Accuracy of recall and reaction times were recorded.


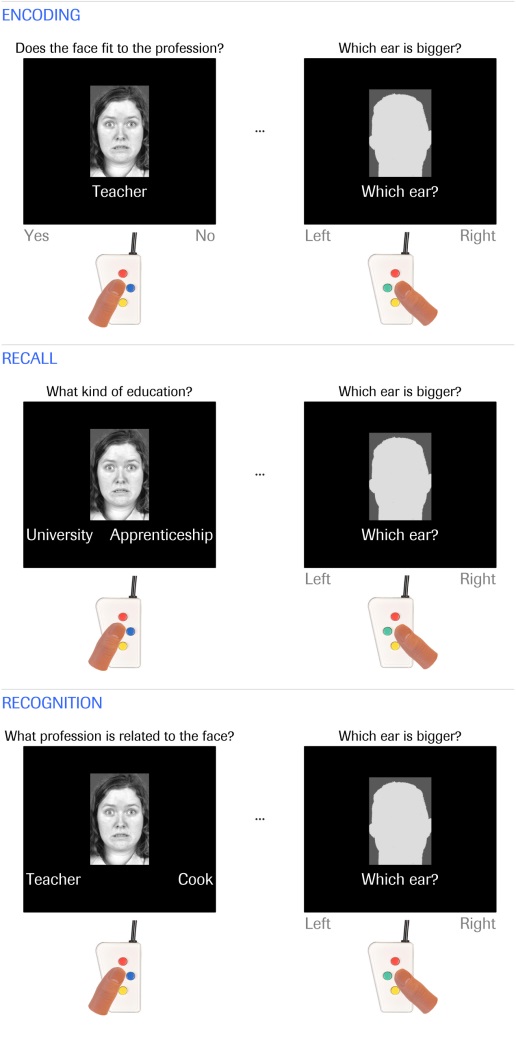


**Fig D.** **Episodic memory task**.

### Theory of Mind

The task was adopted from Schnell et al. ([Schnell et al., 2011](#_ENREF_6)). Thirty-two false-belief cartoon stories were presented. Each story was composed of 3 consecutive pictures (7.53 s/picture) developing a story around a protagonist who was distinguished from other figures in the story by a bold outline (Fig E). For each condition 8 trials consisted of text instructions (6.53 s) and a cartoon story (22.58 s). Participants were instructed to judge picture-to-picture changes in visuospatial (V) representations or affective states (A) from the third person perspective (3rd pp), resulting in a total of 2 conditions: third person visuospatial (3rd ppV) and third person affective (3rd ppA). The different instructions indicating the two conditions were: Judge affective changes from third person perspective (3rdppA): "Does the protagonist feel worse-equal-better compared to the previous picture?”; Judge visuospatial changes from third person perspective (3rdppV): ”Is the number of living beings perceived by the protagonist smaller–equal–greater compared to the previous picture?”. The order of the task conditions was randomized across subjects but fixed within each subject. Participants responded by pressing a button with their right hand: left=less, mid=equal, right=more for visuospatial evaluation, and left=worse, mid=equal, right=better for affective judgment, respectively.


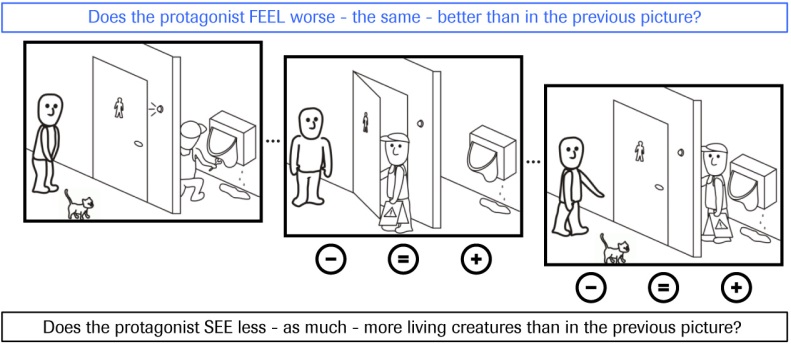


**Fig E.** **Theory of mind task**.

### Go/No-go

A variation of the task of Liddle et al. ([Liddle et al., 2001](#_ENREF_5)) was used (Fig F). Participants were instructed to respond as quickly and accurately as possible by button press every time a Go (‘A’) stimulus was presented and not to respond when a No-go (‘X’) stimulus was presented. Each trial began with a movement preparation phase (1 s) and a pre-cue (250 ms) to heighten preparedness to respond. After the movement preparation phase, the Go/No-go stimulus was presented for 250 ms and the response time is recorded. Reaction time was computed on Go trials for which the participant responded within 1 s post-stimulus. The absence of responses within a period of 1 sec following the onset of a Go stimulus was scored as an error of omission. Responses within 1 s after a No-go stimulus were regarded as errors of commission. The time between the stimulus onset and the end of the trial was jittered (4–6 s). The average trial duration was 6 s. 50 Go and 30 No-go stimuli were presented, resulting in 80 trials.


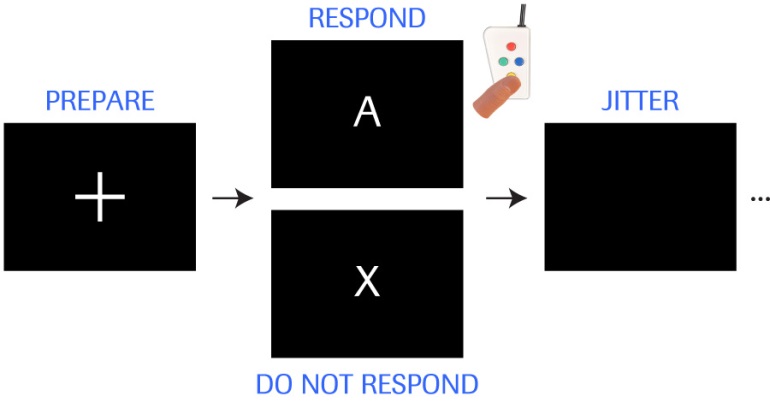


**Fig F.** **Go/No-go task**.

## Supplementary results

**Table B** Behavioral performance during the fMRI tasks.

| **Task** | **Behavioral measure** | **Visit 1 (SD)** | **Visit 2 (SD)** | ***p*-val** | **ICC [LB, UB]** |
| --- | --- | --- | --- | --- | --- |
| **MID** | Mean RT Win1 (ms) | 299.19 (61.14) | 258.41 (19.12) | **0.001** | 0.27 [-0.52, 0.65] |
|  | Mean RT Win2 (ms) | 289.34 (57.72) | 251.11 (17.83) | **0.001** | 0.29 [-0.49, 0.66] |
|  | Mean RT Control (ms) | 312.10 (63.33) | 266.00 (20.45) | **<0.001** | 0.46 [-0.12, 0.75] |
|  | Number of hits (n) | 30.43 (9.36) | 34.03 (1.83) | **0.039** | 0.08 [-0.28, 0.43] |
|  | Reward (€) | 33.27 (10.49) | 37.95 (2.73) | **0.019** | 0.10 [-0.26, 0.44] |
| **Face Matching** | Mean RT Faces (ms) | 1156.43 (241.50) | 1052.40 (193.18) | **0.004** | 0.78 [0.55, 0.90] |
|  | Mean RT Shapes (ms) | 1045.93 (194.35) | 930.90 (158.36) | **0.001** | 0.73 [0.43, 0.87] |
|  | Miss Faces (%) | 0.00 (0.00) | 0.13 (0.73) | 0.326 | 0.00 [-0.36, 0.36] |
|  | Incorrect Faces (%) | 3.87 (18.21) | 0.40 (1.22) | 0.308 | -0.01 [-0.36, 0.35] |
| **N-back** | Mean RT 0-back (ms) | 490.00 (133.67) | 487.31 (250.49) | **0.017** | 0.69 [0.34, 0.85] |
|  | Mean RT 2-back (ms) | 555.00 (312.69) | 487.31 (250.49) | 0.258 | 0.55 [0.04, 0.79] |
|  | Hit 0-back (%) | 99.45 (1.18) | 99.52 (1.02) | 0.787 | 0.24 [-0.13, 0.55] |
|  | Hit 2-back (%) | 82.45 (16.67) | 85.62 (13.57) | 0.261 | 0.52 [0.19, 0.74] |
|  | Miss 0-back (%) | 0.00 (0.00) | 0.00 (0.00) | – | – |
|  | Miss 2-back (%) | 0.97 (1.02) | 1.45 (0.91) | **0.050** | 0.13 [-0.24, 0.47] |
|  | Incorrect 0-back (%) | 0.55 (1.18) | 0.48 (1.02) | 0.787 | 0.24 [-0.13, 0.55] |
|  | Incorrect 2-back (%) | 16.55 (16.44) | 12.93 (13.78) | 0.191 | 0.54 [0.22, 0.75] |
| **ToM** | Mean RT Feel (ms) | 2775.63 (774.24) | 2648.27 (586.52) | 0.143 | 0.87 [0.73, 0.94] |
|  | Mean RT Count (ms) | 2388.80 (684.57) | 2289.50 (555.58) | 0.293 | 0.80 [0.58, 0.91] |
|  | Miss Feel (%) | 26.70 (14.42) | 22.83 (16.81) | 0.209 | 0.45 [0.11, 0.69] |
|  | Miss Count (%) | 58.60 (21.61) | 59.63 (21.57) | 0.846 | 0.10 [-0.26, 0.44] |
|  | Incorrect Feel (%) | 22.00 (16.71) | 21.73 (8.96) | 0.931 | 0.21 [-0.15, 0.53] |
|  | Incorrect Self (%) | 7.77 (8.79) | 6.23 (6.33) | 0.334 | 0.38 [0.03, 0.65] |
| **Go/No-go** | Mean RT (ms) | 392.70 (108.47) | 392.63 (242.06) | 0.999 | 0.32 [-0.43, 0.68] |
|  | Incorrect Go (%) | 0.93 (2.61) | 2.93 (11.37) | 0.348 | 0.03 [-0.33, 0.38] |
|  | Incorrect NoGo (%) | 6.30 (7.31) | 5.07 (4.95) | 0.253 | 0.57 [0.27, 0.77] |
| **Encoding** | Mean RT Professions (ms) | 1793.93 (396.18) | 1612.63 (259.91) | **0.002** | 0.77 [0.51, 0.89] |
|  | Mean RT ears (ms) | 1642.03 (317.01) | 1544.70 (268.90) | **0.046** | 0.77 [0.51, 0.89] |
| **Recall** | Mean RT (ms) | 2330.90 (482.39) | 2219.90 (395.72) | 0.129 | 0.76 [0.49, 0.89] |
|  | Miss (%) | 0.20 (1.10) | 0.80 (2.61) | 0.264 | -0.04 [-0.39, 0.32] |
|  | Incorrect (%) | 27.03 (11.91) | 26.90 (11.09) | 0.960 | 0.20 [-0.17, 0.52] |
| **Recognition** | Mean RT (ms) | 1577.50 (215.66) | 1573.63 (235.38) | 0.943 | 0.26 [-0.55, 0.65] |
|  | Miss (%) | 3.63 (4.99) | 3.00 (3.43) | 0.525 | 0.21 [-0.16, 0.52] |
|  | Incorrect (%) | 13.20 (11.45) | 10.93 (10.62) | 0.174 | 0.67 [0.42, 0.83] |

# ICC(2,k) was used for all average measures, ICC(1,k) was used for all other outcomes, LB and UB – lower and upper bound of the 95% confidence interval

**Table C** Results of tasks specific ROI analyses

| **Task** | **ROI** | **ICC[LB-UB]** |
| --- | --- | --- |
| MID | Bilateral ventral striatum | 0.80 [0.58-0.91] |
| N-back | Bilateral dlPFC | -0.34 [-1.85-0.37] |
| Face Matching | Left amygdala | 0.30 [-0.47-0.67] |
| Face Matching | Right amygdala | 0.18 [-0.72-0.61] |
| Theory of Mind | Bilateral medial PFC | 0.75 [0.48-0.88] |

# ICC(2,k) was used for all ROI reliability analyses, LB and UB – lower and upper bound of the 95% confidence interval

**Table D** Median voxel-wise reliabilities within resting state networks

Median voxel-wise reliability [5-95^th^ %ile] for the respective networks are provided, *ICC(2,1), ALFF – amplitude of low frequency fluctuations, ant – anterior, CBF – cerebral blood flow, DC – degree centrality, ECM – eigenvector centrality mapping, fALFF – fractional amplitude of low frequency fluctuations, LECN and RECN – left and right executive control network, post – posterior, prim – primary, ReHo – regional homogeneity, vDMN and dDMN – ventral and dorsal default mode network.

# Main Effect of Tasks


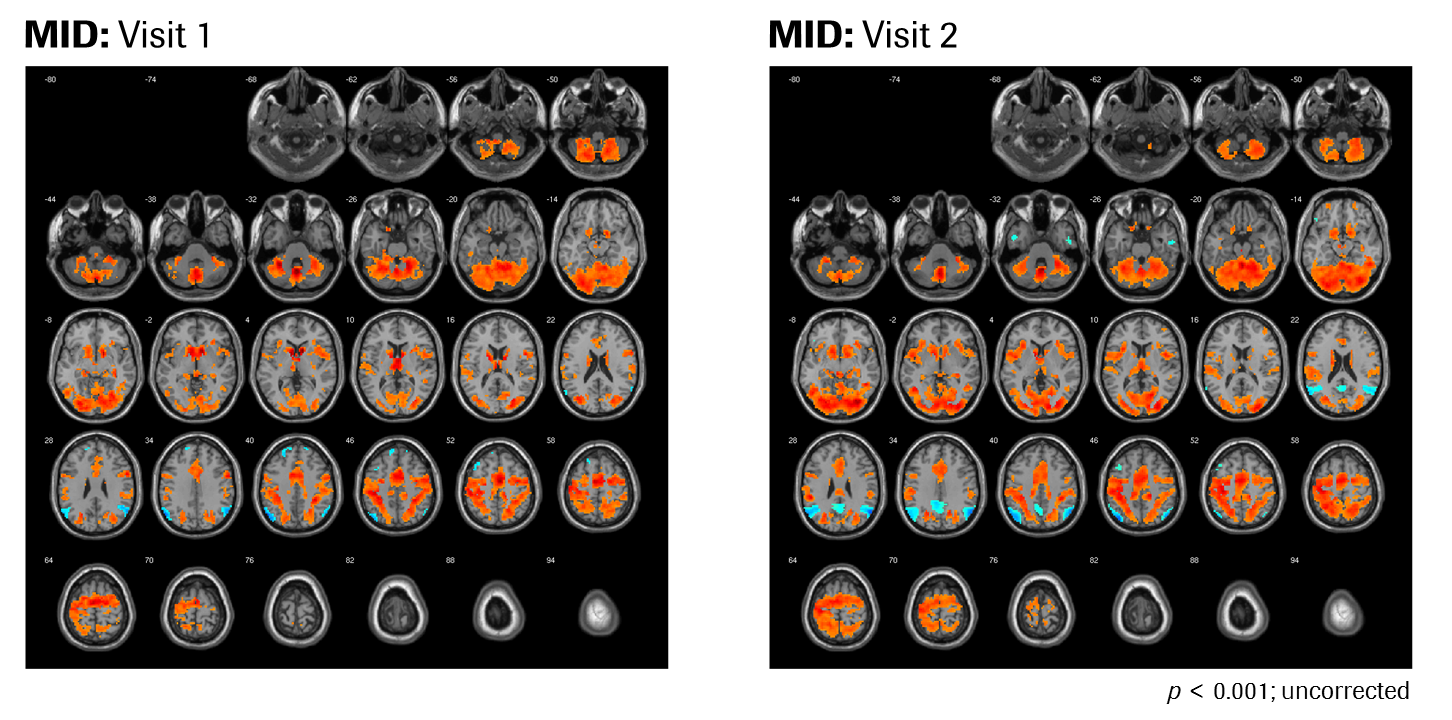


**Fig G.** **Main effect of the MID task at Visit 1 and Visit 2**.


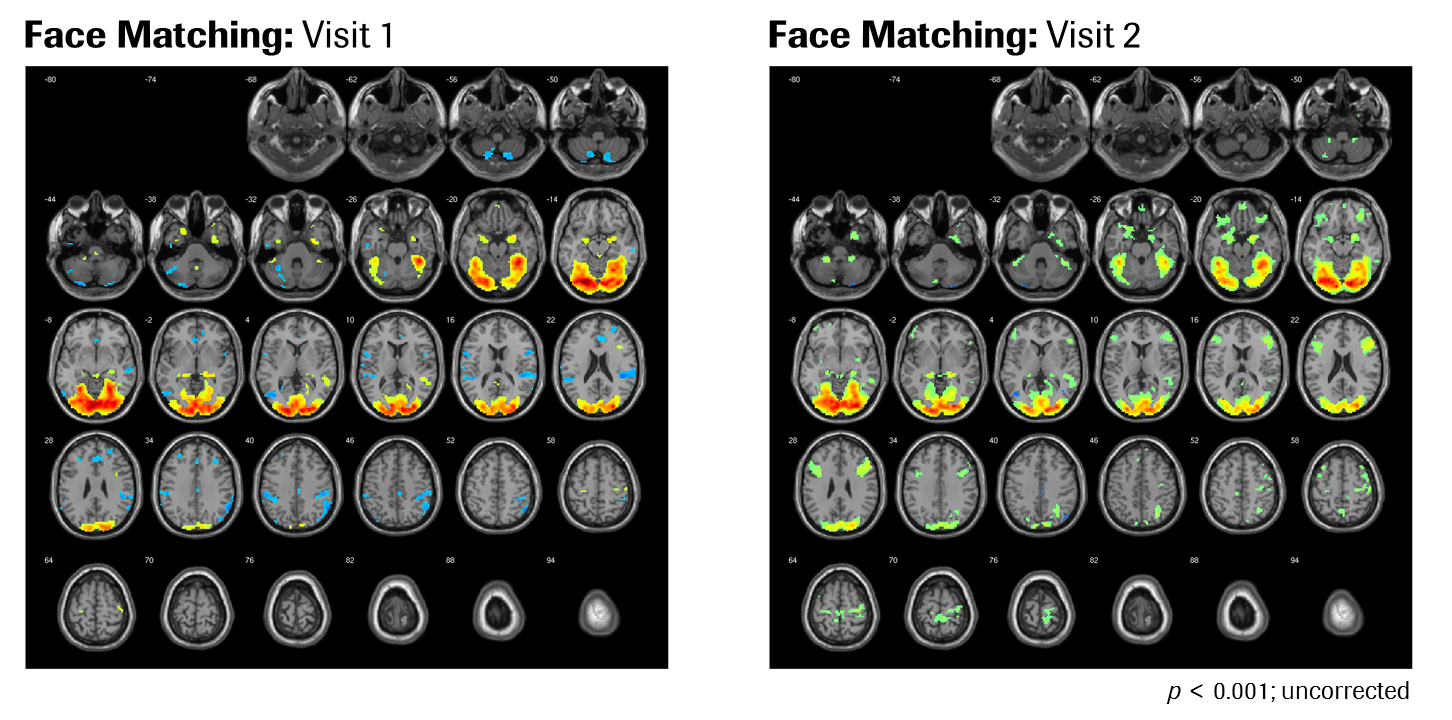


**Fig H.** **Main effect of the Face Matching task at Visit 1 and Visit 2**.


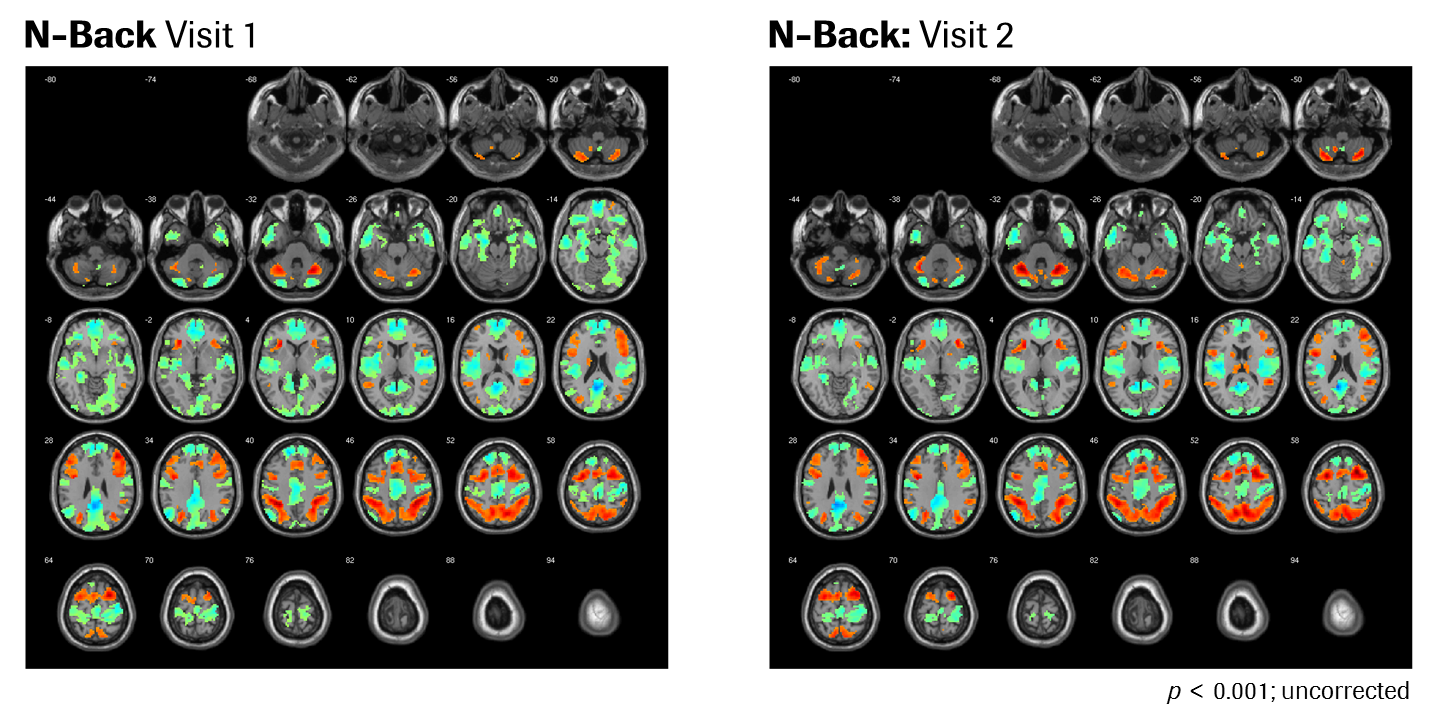


**Fig I.** **Main effect of the N-back task at Visit 1 and Visit 2**.


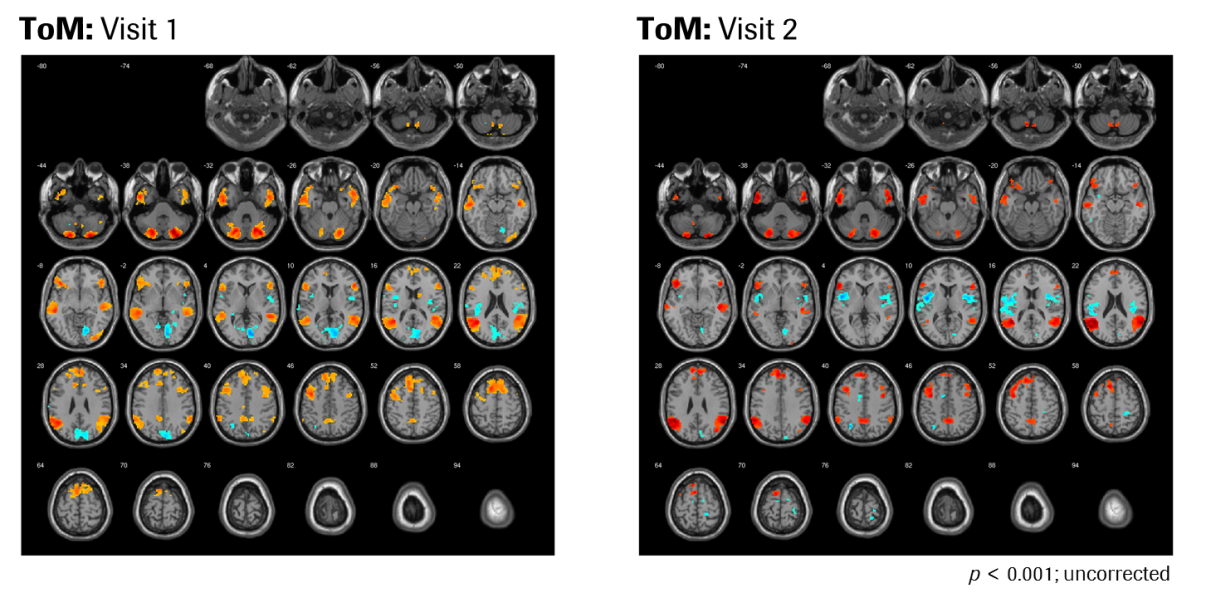


**Fig J.** **Main effect of the ToM task at Visit 1 and Visit 2**.


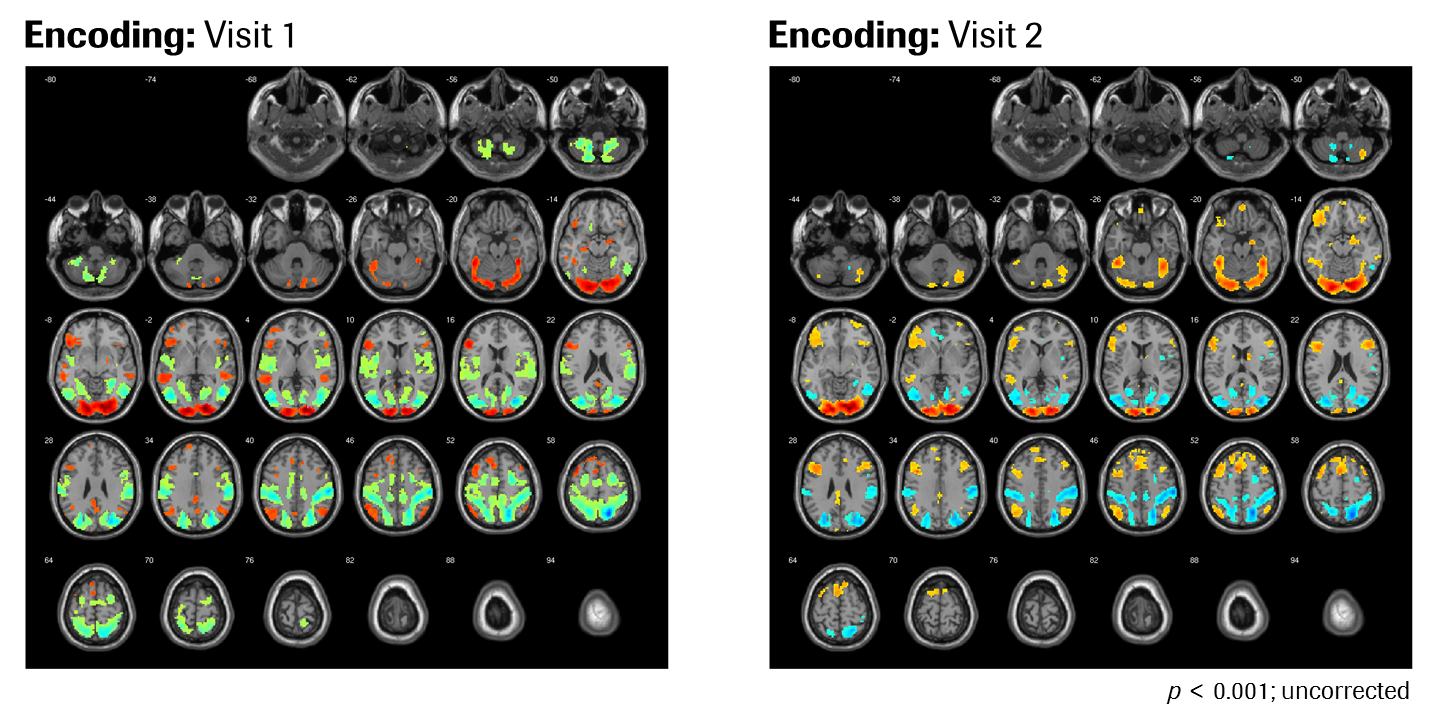


**Fig K.** **Main effect of the Encoding task at Visit 1 and Visit 2**.


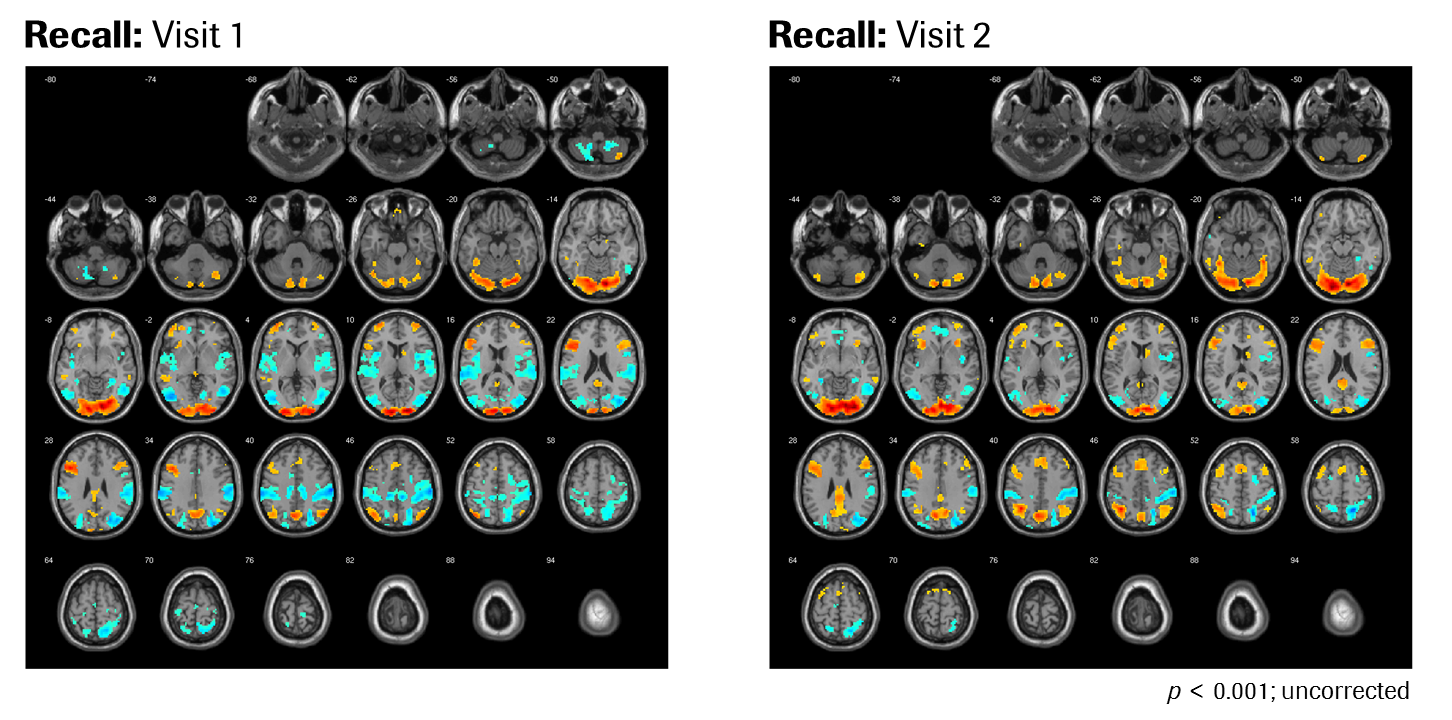


**Fig L.** **Main effect of the Recall task at Visit 1 and Visit 2**.


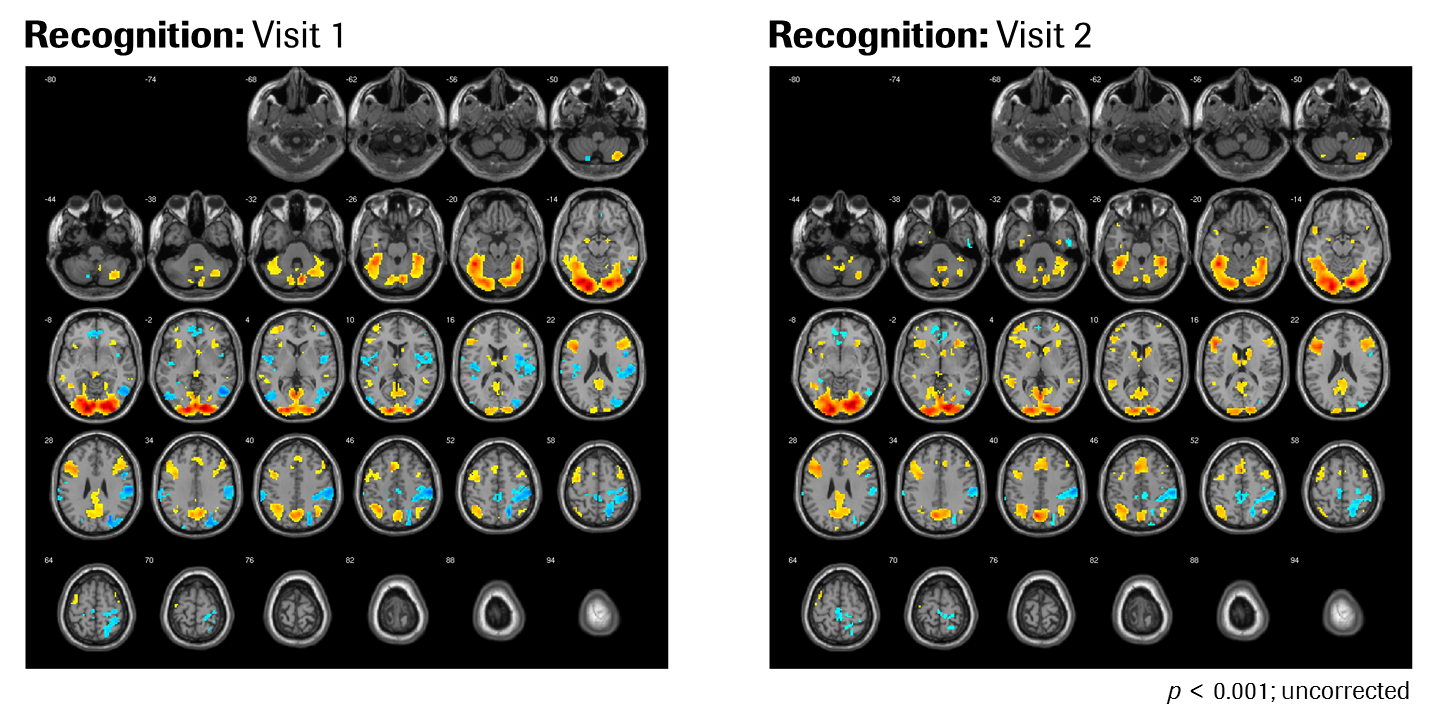


**Fig M.** **Main effect of the Recognition task at Visit 1 and Visit 2**.

# References

Callicott, J.H., Mattay, V.S., Bertolino, A., Finn, K., Coppola, R., Frank, J.A., Goldberg, T.E., Weinberger, D.R., 1999. Physiological characteristics of capacity constraints in working memory as revealed by functional MRI. Cereb Cortex 9, 20-26.

Erk, S., Spottke, A., Meisen, A., Wagner, M., Walter, H., Jessen, F., 2011. Evidence of neuronal compensation during episodic memory in subjective memory impairment. Arch Gen Psychiatry 68, 845-852.

Hariri, A.R., Mattay, V.S., Tessitore, A., Kolachana, B., Fera, F., Goldman, D., Egan, M.F., Weinberger, D.R., 2002. Serotonin transporter genetic variation and the response of the human amygdala. Science 297, 400-403.

Knutson, B., Bhanji, J.P., Cooney, R.E., Atlas, L.Y., Gotlib, I.H., 2008. Neural responses to monetary incentives in major depression. Biol Psychiatry 63, 686-692.

Liddle, P.F., Kiehl, K.A., Smith, A.M., 2001. Event-related fMRI study of response inhibition. Hum Brain Mapp 12, 100-109.

Schnell, K., Bluschke, S., Konradt, B., Walter, H., 2011. Functional relations of empathy and mentalizing: an fMRI study on the neural basis of cognitive empathy. Neuroimage 54, 1743-1754.
